# Numerical assessment of the applicability of geometry-based force inference on homogeneous and heterogeneous cells

**DOI:** 10.1371/journal.pone.0299016

**Published:** 2024-04-16

**Authors:** Shou Miyasaka, Keita Izumi, Satoru Okuda, Yuichiro Miki

**Affiliations:** 1 Nikon Corporation, Tokyo, Japan; 2 WPI-Nano Life Science Institute, Kanazawa University, Kanazawa, Japan; University of Vigo, SPAIN

## Abstract

The measurement of cellular forces, which reflect crucial biological attributes, has the potential to replace conventional cell assessment methods, such as morphology, proliferation, and molecular expression analysis, in medical cell diagnosis and cell culture studies. In medical cell evaluations, force inference techniques have gained prominence due to their non-invasiveness and lack of requirement for specialized equipment. Among those techniques, the method proposed by Ishihara et al., which estimates forces in densely packed cells based only on cell geometry, is a promising method. However, its applicability range of this method has not been fully established. In this study, we employed a two-dimensional vertex model to numerically assess the applicability of this method on homogeneous and heterogeneous cells. Our comparisons between the true values from numerical simulations and the estimated values from the inference method revealed a significant correlation between estimation accuracy and cell roundness in systems of homogeneous cell. Moreover, the method demonstrated efficient force estimations in heterogeneous-cell systems. These findings may be useful when the force inference method is employed to evaluate medical cells.

## Introduction

In medical cell diagnosis and cell culture studies, intracellular forces, which reflect biological properties, have the potential to replace conventional cell assessment methods, such as morphology, proliferation, and molecular expression analysis. For instance, the mechanical properties of cells can be used to identify senescent cells, which are characterized by a stable cell cycle arrest induced in response to stress [1]. This chronic inflammatory state fosters a pro-tumorigenic microenvironment, promoting cancer initiation, migration, and metastasis. The in vivo detection of senescence necessitates the examination of fixed or deep-frozen tissues, as in the immunohistochemical analysis of frozen samples [2]. However, there is considerable clinical demand for real-time bioimaging techniques. Senescent cells exhibit enhanced mechanical maturity at adhesion points, leading to the transmission of greater traction forces to the substrate. Consequently, the detection of senescent cells can be achieved by observing alterations to their cell morphology [3] or quantifying the mechanical forces they generate. There are numerous other instances where the state of a cell and the mechanical stress exerted on it are closely related [4, 5].

Many approaches have been proposed for investigating the mechanical properties of cells. These approaches can be divided into two types: one involves applying force directly to cells and measuring the amount of cell deformation as an equivalent of the force on a cell [5, 6], while the other involves non-invasive measuring physical or chemical indices that are indirectly related to cellular forces [7, 8]. Despite the efficacy of these methods, their invasiveness, expense associated with the preparation of specialized platforms, and limited throughput pose substantial challenges for practical applications, such as cell assessment [9].

In order to address these challenges, numerical inference methods have attracted attention. Such methods estimate tension at cell-cell boundaries and intracellular pressure under the assumption of force equilibrium among cells [9–12]. Specifically, the approach proposed by Ishihara et al. [9] uses Bayesian statistics to deal with the indeterminacy inherent in the estimation process [9, 10, 13, 14]. This non-invasive technique does not require specialized equipment and can be readily integrated with a conventional microscope. However, its applicability to actual cell evaluation has limitations. The validation of this method has focused on a limited set of parameters for homogeneous cells using *Drosophila* wing cells as a model. Thus, to expand its application to cells utilized in medical cell evaluation, such as human cells, it is essential to widen the scope of validation.

In this study, we investigate the applicability of the force inference method proposed by Ishihara et al. [9]. To achieve this, we apply the method to the cell morphologies derived from numerical simulations using a two-dimensional (2D) vertex model and assess the dependence of estimation accuracy on cell behavior by comparing simulated and estimated forces. Furthermore, by analyzing the correlation between estimation accuracy and cell morphology, we identify the conditions under which the inference method has high accuracy. Based on these results, we discuss the applicability of the inference method for homogeneous and heterogeneous cells.

## Methods

To assess the applicability of the force inference method proposed by Ishihara et al. [9], we conducted numerical simulations utilizing a 2D vertex model and then applied the inference method to the cell morphologies derived from the model. In this approach and model, cells are presumed to be densely packed and are represented as simplified polygonal shapes with straight edges. An overview of the force inference method and a description of the 2D vertex model are given in the following sections.

### Force inference

**Fig 1:**
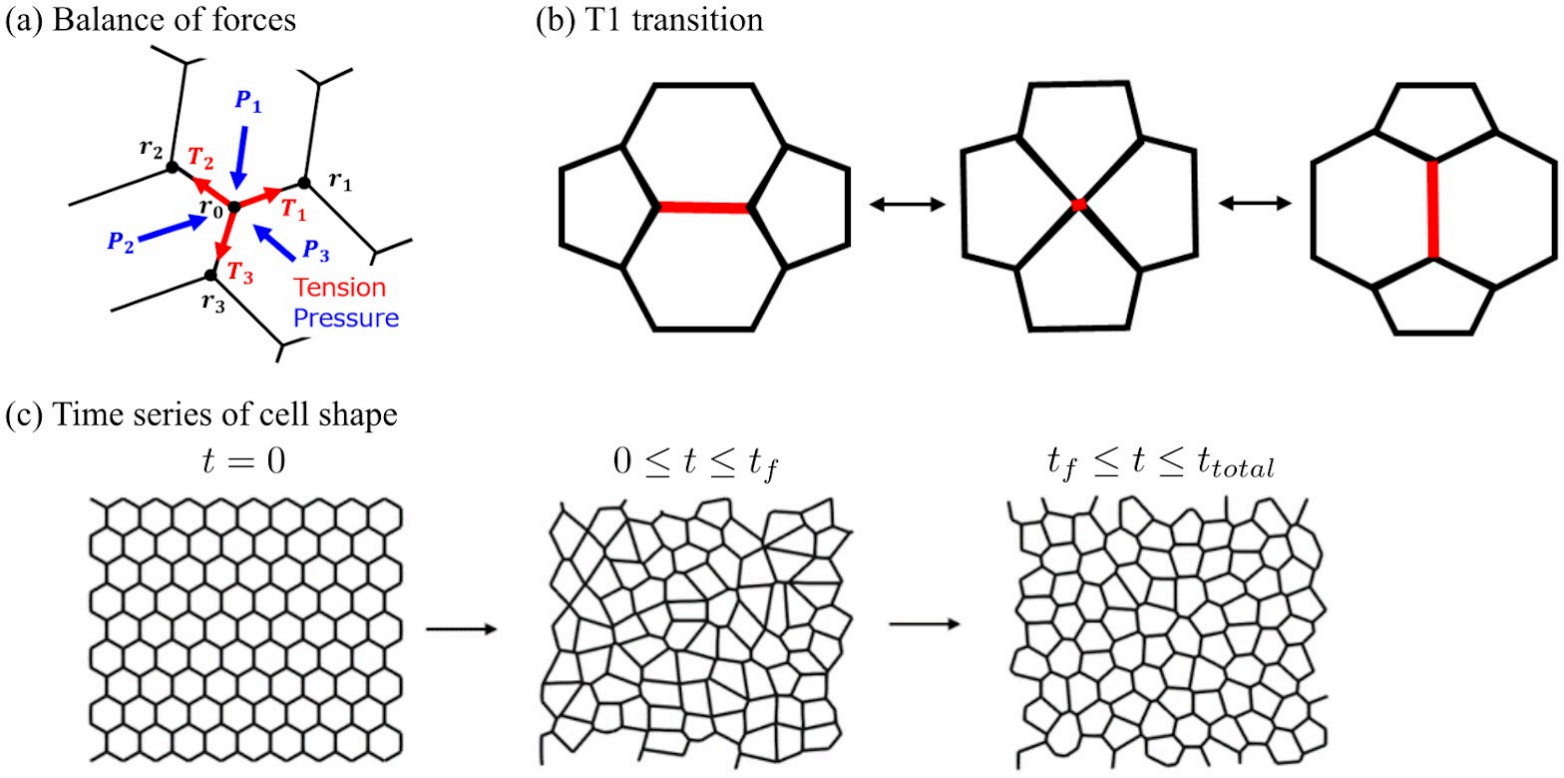
Numerical simulation using a 2D vertex model. (a) Diagram of forces around vertex in 2D vertex model. Blue arrows represent intracellular pressure toward the vertex and red arrows represent tension at cell-cell boundaries. (b) Illustration of T1 transition implemented in 2D vertex model. The left and right cells approach each other. When the length of the edge shown in red becomes shorter than a certain threshold, the cells acquire a common edge and top and bottom cells separate. (c) Flow of 2D vertex model. In the period 0 ≤ *t* ≤ *t*_*f*_ the system is relaxed by adding a fluctuation term to the line tension. In the period *t*_*f*_ ≤ *t* ≤ *t*_*total*_, the system is transformed to a steady state to minimize energy by removing the fluctuation term.

The force inference method estimates the tension at cell-cell boundaries and intracellular pressure by solving the force balance equation at each vertex (Fig 1(a)). The position vector of the *i*-th vertex is denoted by ***r***_*i*_. If there are *ni* vertices connected to the *i*-th vertex through edges, the forces acting on the *i*-th vertex in the *x* and *y* directions are given by

$$\begin{array}{c} f_{i}^{x}=\sum_{j=1}^{n_{i}}\frac{x_{j}-x_{i}}{\left|r_{j}-r_{i}\right|}T_{j}+\sum_{j=1}^{n_{i}}\frac{y_{\left(j\;\text{mod}\;n_{i}\right)+1}-y_{j}}{2}P_{j},\\ f_{i}^{y}=\sum_{j=1}^{n_{i}}\frac{y_{j}-y_{i}}{\left|r_{j}-r_{i}\right|}T_{j}+\sum_{j=1}^{n_{i}}\frac{x_{\left(j\;\text{mod}\;n_{i}\right)+1}-x_{j}}{2}P_{j}, \end{array}$$ (1)

where *i* identifies the vertex, *T*_*j*_ is the tension on the *j*-th edge, and *P*_*j*_ is the pressure of the *j*-th cell adjacent to both the *j*-th and (*j* + 1)-th edges. Considering Eq (1) for all vertices in the system, the vector **F** (= (*f*_1_^*x*^, *f*_1_^*y*^, *f*_2_^*x*^, *f*_2_^*y*^, …)) containing all *xy* elements of the forces can be written as

F=AS, (2)

where **S** (= (*T*_1_, *T*_2_, …, *P*_1_, *P*_2_, …)) is the matrix that summarizes the edge tension and cell pressure to be estimated and **A** is the matrix that summarizes the coefficients that reflect cell morphologies. Since the cell deformation process is quasi-static under the low Reynolds number assumption, the tensions and pressures are balanced at each vertex. Thus, the force balance equation can be formulated as

AS=0. (3)

A Bayesian estimation technique is used to solve Eq (3). Specifically, the prior function is assumed to be a Gaussian distributed around some positive tension value. The hyperparameter, which represents the ratio of the variance of the prior function to that of the likelihood, is calculated by maximizing the marginal likelihood. The estimation of **S** is then accomplished by maximizing the posterior distribution. Further details regarding this inference method are described in previous studies [9, 10].

### Acquisition of stress field using 2D vertex model

To evaluate the accuracy of the force inference method, numerical simulations were conducted using a 2D vertex model [15]. The simulations were performed on a system that contained 100 cells confined within a box measuring 10 units in the *x* and *y* directions. Periodic boundary conditions were applied to all boundaries. The motion of each cell was expressed through vertex movements under quasi-static conditions and rearrangements between cells were expressed through the T1 transition (Fig 1(b)) by reconnecting edge connections based on vertex movements [16]. The reconnection was performed when the edge length became shorter than the threshold, *l*_*T*1_ = 0.05, a value chosen to be small enough to affect the calculation of cell morphology. In the vertex model, the cell morphologies were determined by minimizing the potential energy of the system. The cellular network was sufficiently relaxed before the calculation to avoid local minima. In this section, the validation of the force inference method using the simulation results and details of the simulation procedure are discussed first, and the applied parameter settings are presented later.

In the static state, the mechanical force balance of cell configurations can be represented by a potential energy function [17]. The potential energy is defined as

$$U\equiv \sum_{i}^{\text{cell}}\frac{K}{2}{\left(s_{i}-s_{\text{eq}}\right)}^{2}+\sum_{i}^{\text{cell}}\frac{\Gamma_{i}}{2}p_{i}^{2}+\sum_{j}^{\text{edge}}\Lambda_{j}l_{j},$$ (4)

where *s*_*i*_ is the area of the *i*-th cell, *p*_*i*_ is the perimeter of the *i*-th cell, *l*_*j*_ is the length of the *j*-th edge, the first term represents the area elasticity, the second term represents the perimeter elasticity, and the third term represents the line tension. The area elastic modulus *K*, the preferred area *s*_eq_, and the perimeter elasticity *Γ*_*i*_ are parameters that determine the mechanical behavior of the system. The perimeter elasticity *Γ*_*i*_ is randomly assigned to each cell according to a Gaussian distribution with mean *μ*_*Γ*_ and standard deviation *σ*_*Γ*_. The line tension *Λ*_*j*_ is affected by the actin-myosin contractile force at cell-cell boundaries. The pressure of the *i*-th cell and the tension at the *j*-th edge in the static state are calculated as

$$\begin{array}{c} P_{i}=-\frac{\partial U}{\partial \text{s}}=-K\left(s_{i}-s_{eq}\right)\\ T_{j}=-\frac{\partial U}{\partial l}=\Gamma_{i}p_{i}+\Gamma_{i+1}p_{i+1}+\Lambda_{j}, \end{array}$$ (5)

where *p*_*i*_ and *p*_*i*+1_ are the perimeters of the *i*-th and (*i* + 1)-th cells, including the *j*-th edge, respectively.

To compare the estimated values with the true values, the true and estimated tension values were scaled by their respective scaling factors in accordance with a previous study [9]. For example, for the estimated values, the scaling factor, denoted by *c*, was determined as $c=1/\bar{T_{\text{est}}}$, where $\bar{T_{\text{est}}}$ is the mean value of the estimated tension. The scaling factors were chosen such that the average tension values were equal to 1. The true and estimated pressures were scaled using the same factor *c* to ensure that the average pressure values were 0; that is, ${\tilde{P}}_{est}=cP_{est}+\Delta p$, where $\Delta p=-c\bar{P_{est}}$ and $\bar{P_{est}}$ is the mean value of the estimated pressure. Using the scaled true and estimated values, we calculated the estimation accuracy in terms of the root-mean-squared error (RMSE) *σ*_*est*_ as

$$\sigma_{\text{est}}\equiv \sqrt{\frac{\sum_{i}^{n_{\text{cell}}}{\left({\tilde{P}}_{\text{est}\;i}-{\tilde{P}}_{\text{true}\;i}\right)}^{2}+\sum_{j}^{n_{\text{edge}}}{\left({\tilde{T}}_{\text{est}\;j}-{\tilde{T}}_{\text{true}\;j}\right)}^{2}}{n_{\text{cell}}+n_{\text{edge}}}},$$ (6)

where ${\tilde{P}}_{est\;i}$ and ${\tilde{P}}_{true\;i}$ are the scaled estimated and true pressures, respectively, for the *i*-th cell, *n*_cell_ is the number of cells, ${\tilde{T}}_{est\;j}$ and ${\tilde{T}}_{true\;j}$ are the scaled estimated and true tensions, respectively, for the *j*-th edge, and *n*_edge_ is the number of edges.

The cell morphology and force in the static state were obtained by calculating vertex movements:

$$\eta \frac{dr_{i}}{dt}=-\nabla U,$$ (7)

where *η* is the friction coefficient. The numerical integration of Eq (7) was conducted using the first-order Euler method with time step *Δt*. Topological reconnection of edges was carried out when the edge length was less than the threshold value, *l*_*T*1_.

To obtain a system state with potential energy near the global minimum, simulations using the annealing method were performed. Two distinct processes were carried out in sequence. First, a fluctuation process was calculated, in which the fluctuation of the line tension *Λ*_*j*_ in Eq (8) was incorporated during the period 0 ≤ *t* ≤ *t*_*f*_. Second, a relaxation process was calculated, in which the fluctuation was gradually reduced during the period *t*_*f*_ < *t* ≤ *t*_total_ (Fig 1(c)). The line tension *Λ*_*j*_ is written as

$$\Lambda_{j}=\left\{\begin{array}{cc} \Lambda_{j}^{c}+\omega_{j} & \text{if}\;\;\;0\leq t\leq t_{f}\\ \Lambda_{j}^{c}+\omega_{j}\text{exp}\left(-t\right) & \text{if}\;t_{f}<t\leq t_{\text{total}} \end{array}\right.,$$ (8)

where the constant $\Lambda_{j}^{c}$ represents the actin-myosin contractile force. Its value varied across cell-cell boundaries according to a Gaussian distribution, $\Lambda_{j}^{c}\sim N\left(\mu_{\Lambda },\;\sigma_{\Lambda }^{2}\right)$, where *μ*_*Λ*_ and *σ*_*Λ*_ denote the mean and standard deviation, respectively. The variable *ω*_*j*_ is colored noise with time correlation. Its time evolution is given by

$$\frac{d\omega_{j}\left(t\right)}{dt}=-\frac{1}{\tau_{j}}\omega_{j}\left(t\right)+\xi_{j}.$$ (9)

where ξ_*j*_ is white noise according to a Gaussian distribution, $\xi_{j}\sim N\left(0,\;\sigma_{f}^{2}/\tau_{f}^{2}\right)$, where *σ*_*f*_ and *τ*_*f*_ denote the amplitude and correlation time of *ω*_*j*_, respectively [18, 19].

Table 1 presents the physical and numerical parameters utilized in the simulations using the 2D vertex model. The unit length was set to $\sqrt{s_{eq}}$, the unit energy was set to ${Ks}_{eq}^{2}$, and the unit time was set to *η*⁄*K*. The parameters *Γ*, *μ*_*Γ*_, *σ*_*Γ*_, and *μ*_*Λ*_ are control parameters that reflect a heterogeneous cellular system; their respective ranges are shown in Table 2.

**Table 1:** List of constants and variables used in 2D vertex model.

Parameter	Description	Set value	Unit
*dt*	Time step	0.01	*η*⁄*K*
*t*_*total*_	Total simulation time	2000	*η*⁄*K*
*t*_*in*_	Time interval for intercalation	50	*η*⁄*K*
*s*_*eq*_	Ideal area	1.0	1
*l*_0_	Length of one side of initial cell	0.62	$\sqrt{s_{eq}}$
*p*_*eq*_	Ideal perimeter	3.7	$\sqrt{s_{eq}}$
*l*_*T*1_	Limit length for T1 transition	0.05	$\sqrt{s_{eq}}$
*C*_*T*1_	Correlation factor of length after T1 transition	1.5	–
*η*	Friction coefficient of vertex	1.0	1
*K*	Area elastic modulus	1.0	1
*Γ*	Perimeter elasticity	Control	*Ks*_*eq*_
*μ*_*Γ*_	Mean of perimeter elasticity	Control	*Ks*_*eq*_
*σ*_*Γ*_	Standard deviation of perimeter elasticity	Control	*Ks*_*eq*_
*Λ*	Line tension	Control	*K*(*s*_*eq*_)^3 ⁄ 2^
*μ*_*Λ*_	Mean of constant tension term *Λ*^*c*^	Control	*K*(*s*_*eq*_)^3 ⁄ 2^
*σ*_*Λ*_	Standard deviation of constant tension term *Λ*^*c*^	0.05	*K*(*s*_*eq*_)^3 ⁄ 2^
*t*_*f*_	Term used to set thermal fluctuation	1000	*η*⁄*K*
*τ*_*f*_	Relaxation time of tension fluctuation term *ω*	20.0	*η*⁄*K*
*σ*_*f*_	Coefficient in fluctuation term	10.0	*K*(*s*_*eq*_)^3 ⁄ 2^

**Table 2:** Typical ranges of parameters with “Control” as set value in Table 1.

Parameter	Range
*Γ*	0.05–0.20
*μ*_*Γ*_	0.05–0.20
*μ*_*Λ*_	-1.4–0.5
*σ*_*Λ*_	0.01–0.04

## Results

To comprehensively investigate the applicability of the force inference method, we conducted two distinct analyses. First, we assessed the accuracy of the estimated cell behavior in a system with homogeneous cells. Numerical simulations using the 2D vertex model were performed to obtain cell morphologies, as well as the tension at cell-cell boundaries and cell pressure, within a wide range of parameter values. The force inference method was then applied to the simulated cell morphologies to estimate the tension and cell pressure. In addition, estimation accuracy was calculated for each parameter set by comparing the forces obtained from the simulations and estimations. Second, we examined the applicability of the inference method to a system with heterogeneous cells.

### Dependence of estimation accuracy on cell behaviors

**Fig 2:**
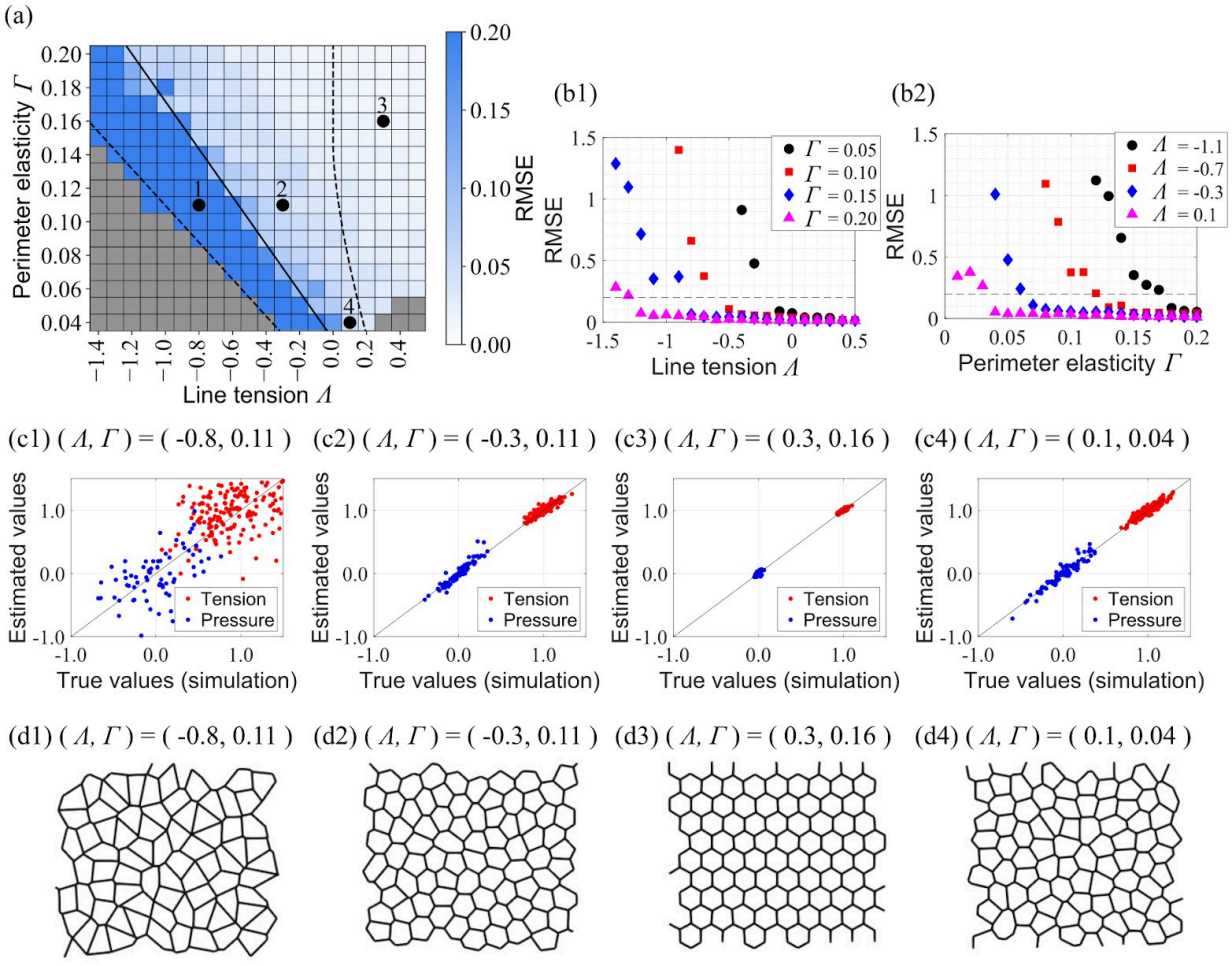
Parameter dependence of estimation accuracy in systems with homogeneous cells. (a) Heatmap of RMSE in 2D parameter space of parameter elasticity and line tension (*Λ*, *Γ*). Gray cells indicate parameter sets for which the simulation stopped due to a large distortion of cell morphology. (b1, b2) Dependence of estimation accuracy on *Λ* and *Γ*. Dashed line shows the threshold of high-accuracy estimation. (c1-c4) Scatter plots of estimated values and true values at four representative points (1–4) indicated in (a), namely (*Λ*, *Γ*) = (−0.8, 0.11), (*Λ*, *Γ*) = (−0.3, 0.11), (*Λ*, *Γ*) = (0.3, 0.16), and (*Λ*, *Γ*) = (0.1, 0.04). The parameter set (*Λ*, *Γ*) = (0.1, 0.04) was used by Ishihara et al. (2012) for verifying their technique. All edge tensions (red) and cell pressures (blue) are plotted. (d1-d4) Cell morphology for four conditions simulated using 2D vertex model.

First, the inference method was applied to the cell morphologies obtained from numerical simulations of homogeneous cells. Fig 2(a) shows the parameter dependence of estimation accuracy in terms of the RMSE defined in Eq (6), where a smaller value indicates higher accuracy. The heat map in the figure shows that the accuracy increases with increasing perimeter elasticity and line tension. The RMSE as a function of *Λ* and *Γ* is plotted in Fig 2(b1) and 2(b2), respectively; the RMSE increases nonlinearly with decreasing either *Λ* or *Γ*. We define the condition with an RMSE of 0.2 or less as the high-accuracy condition, corresponding to the parameter region above the solid line in Fig 2(a). The threshold of RMSE is defined as the result of two-segmented linear regression applied to the plots in Fig 2(b1) and 2(b2) as follows. The points within each of those plots are divided into two groups based on a specific value of *Λ* or *Γ*. For each group, a regression line is obtained using the least squares method, and the grouping is performed to minimize the sum of the residuals of these regression lines. The RMSE value of the intersection point of these two regression lines is extracted for the plot. This process is carried out for all the plots in Fig 2(b1) and 2(b2), and the largest RMSE value is defined as the threshold.

### Correlation between estimation accuracy and characteristics of cell morphology

We examined the relationship between cell morphology and estimation accuracy by analyzing the dependence of cell morphology on the line tension *Λ* and perimeter elasticity *Γ*. Following a previous study [17], we divided the parameter space into three regions based on the ground state of the energy function (Eq (4)), as shown by the dashed line in Fig 2(a). We then compared cell morphology and estimation accuracy (Fig 2(d1)–2(d4)) as well as the true and estimated force values under four typical conditions, namely (*Λ*, *Γ*) = (−0.8, 0.11) (Fig 2(c1)), (*Λ*, *Γ*) = (−0.3, 0.11) (Fig 2(c2)), (*Λ*, *Γ*) = (−0.3, 0.16) (Fig 2(c3)), and (*Λ*, *Γ*) = (0.1, 0.04) (Fig 2(c4)). For the first condition, where the estimation accuracy is low, the cell shapes tend to be elongated and have multiple configurations that can form at the energy minimum. In contrast, for the other conditions, where the estimation accuracy is relatively high, the cell shapes tend to be relatively round.

**Fig 3:**
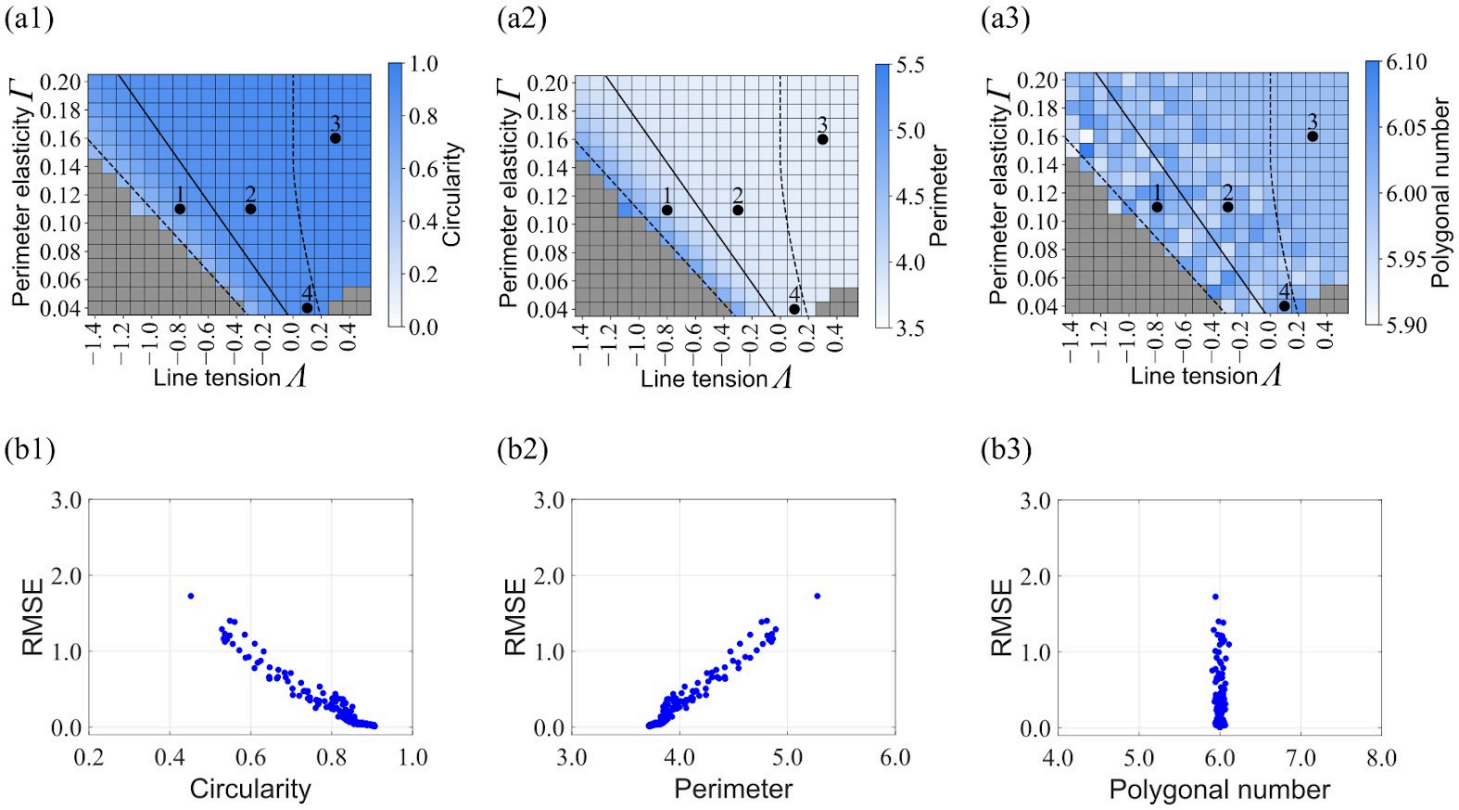
Parameter dependence of cell shape characteristics and correlation with estimation accuracy. (a1-a3) Heatmap of cell shape characteristics (circularity, perimeter, and polygonal number) in 2D parameter space of parameter elasticity and line tension (*Λ*, *Γ*). Solid line is the threshold (RMSE = 0.2) and dashed lines divide the parameter region defined in a previous study [17]. Points 1–4 correspond to representative points in Fig 2(a). (b1-b3) Scatter plots of RMSE versus cell shape characteristics obtained by comparing (a1-a3) and Fig 2(a).

For a more quantitative understanding of the relationship, we computed several characteristic cell morphologies for each parameter set (Fig 3(a1)–3(a3)) and compared them with the estimation accuracy (Fig 3(b1)–3(b3)). The dependence of circularity (4*πs*⁄*p*^2^) on the line tension *Λ* and perimeter elasticity *Γ* is shown in Fig 3(a1). The circularity increases with increasing either *Λ* or *Γ*. By comparing this heatmap and Fig 2(a), we obtained the circularity dependence of estimation accuracy (Fig 3(b1)). For the scatter plot, the regression line is obtained using the least squares method, and the circularity value of the intersection between this regression line and the line of the RMSE threshold is defined as the circularity threshold: 0.82. Moreover, the results for the perimeter are shown in Fig 3(a2) and 3(b2). As a result, the correlation between the perimeter and accuracy is opposite to that of the correlation between circularity and accuracy. This is because a larger circularity generally results in a smaller perimeter, based on the definition of circularity (4*πs*⁄*p*^2^). Furthermore, the results for the polygonal number are plotted in Fig 3(a3) and 3(b3), where no correlation with accuracy is observed.

### Estimation accuracy for heterogeneous cells

**Fig 4:**
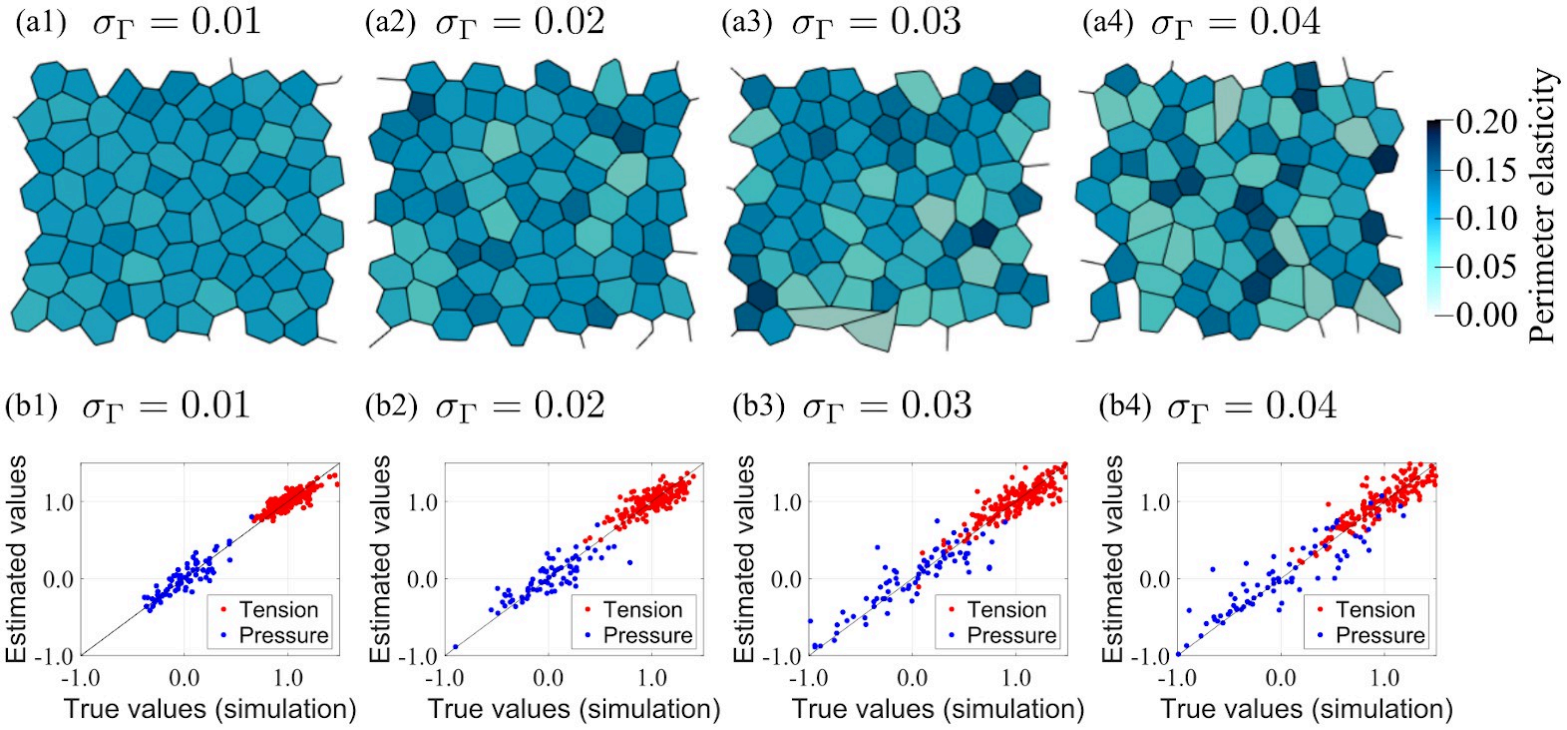
Cell morphology and estimation accuracy in systems with heterogeneous cells. (a1-a4) Cell morphology calculated using 2D vertex model with perimeter elasticity having Gaussian distribution (expressed by color contour). The results were obtained under the conditions where the standard deviation of the perimeter elasticity is set as *σ*_*Γ*_ = 0.01, 0.02, 0.03, and 0.04. (b1-b4) Scatter plots of true and estimated values for each cell morphology.

**Fig 5:**
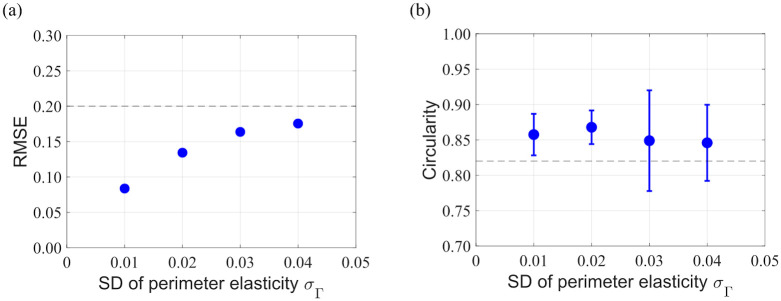
Dependence of estimation accuracy and circularity on cell heterogeneity. (a) Dependence of estimation accuracy on the standard deviation of perimeter elasticity *σ*_*Γ*_ for system with heterogeneous cells. Dashed line is the RMSE threshold (0.2). (b) Circularity for each analysis condition. Points represent the mean circularity value and error bars represent its standard deviation. Dashed line is the circularity threshold (0.82). SD: standard deviation.

To investigate the applicability of the force inference method to systems with heterogeneous cells, we conducted numerical simulations for various values of perimeter elasticity of individual cells and then applied the inference method to the resulting cell morphologies. The cell morphologies are shown in Fig 4(a1)–4(a4), where the color contours indicate the perimeter elasticity *Γ* of each cell. It is observed that cells with a lower perimeter elasticity tend to have a larger area. Scatter plots of the estimated and simulated force values are shown in Fig 4(b1)–4(b4). As *σ*_*Γ*_ increases, the dispersion of tension and pressure also increases; however, the estimated values are close to the true values even for large values of *σ*_*Γ*_. Fig 5(a) shows the RMSE for each analysis. As shown in this plot, the RMSE increases with increasing perimeter elasticity *σ*_*Γ*_. Nonetheless, the RMSE values for all four analyses remain below the threshold (RMSE < 0.2), indicating the possibility of estimating forces with high accuracy, at least within the heterogeneous range of *σ*_*Γ*_ < 0.4. The distribution of circularity for each analysis is shown in Fig 5(b). The average circularity slightly decreases and its variance increases with increasing perimeter elasticity *σ*_*Γ*_. All circularity values are either higher than or comparable to the circularity threshold (dashed line in the figure).

## Discussion

In this study, we employed a two-dimensional vertex model to numerically assess the applicability of the force inference method proposed by Ishihara et al. [9] to systems with homogeneous and heterogeneous cells. The parameter map in Fig 2(a) provides a visual aid for understanding the applicability of this method. To utilize this map, the perimeter elasticity and line tension of the observed cells must be obtained. These parameters can be derived by comparing simulated cell morphologies with actual cell shapes, as demonstrated in previous research on *Drosophila* cells [16, 17]. Our results are consistent with these earlier findings; the parameters for *Drosophila* cells fall within the high-precision range found in our study. Moreover, cell morphology can serve as an index of applicability. We found that when cell circularity exceeds 0.82, high-accuracy estimation is achieved. This suggests that researchers can assess applicability based on cell circularity, which can be calculated using standard microscopy software such as NIS-Elements (Nikon Instruments, Tokyo, Japan).

Our findings also indicate that this inference method is applicable to heterogeneous cell systems with a relative standard deviation of perimeter elasticity below 40%. This is due to the RMSE values falling below the established threshold (RMSE < 0.2). Circularity is also a useful index of applicability to heterogeneous cell systems, as demonstrated in Fig 5(b) (circularity values above a threshold of 0.82). Under actual biological conditions, the mechanical and biochemical properties of cells may vary for a given cell type due to factors such as individual characteristics, the cell cycle, and apoptosis. In our study, we assumed that each cell has a distinct actin cytoskeleton and that perimeter elasticity has a Gaussian distribution. Our results show that the inference method is effective for systems that resemble actual biological conditions. By modifying other factors (e.g., ideal cell area and initial edge length), we can further explore its applicability to heterogeneous systems.

The force inference method has the potential to replace conventional cell evaluation techniques. In medical cell diagnosis and cell culture studies, for example, the mechanical properties of cells could be used to identify senescent cells, which can be distinguished based on altered cell morphology [3]. Given that the estimation method is accurate even for systems with heterogeneous cells, it may be possible to calculate the force exerted on individual cells and distinguish senescent cells from healthy ones based on variations in applied force. If the forces between the two cell types significantly differ, the force distribution may become bimodal. As the prior distribution of Bayesian estimation is linked to force distribution, a potential avenue for future research is to update the prior distribution to further expand the applicability of the inference method. In this study, numerical simulations were used to validate and demonstrate the applicability of the inference method in heterogeneous cells systems, as described above. In order to establish its applicability to actual cells, it is necessary to quantitatively measure the forces acting between heterogeneous cells, and experimental verification is needed in the future.

## Conclusion

Our study demonstrated the potential application of the force inference method proposed by Ishihara et al. [9] to heterogeneous cells systems using a 2D vertex model. Numerical simulations showed the effectiveness of this method in estimating forces for systems with either homogeneous or heterogeneous cells. We also showed the assessment of its applicability using cell circularity. Although we did not apply the method to actual medical diagnosis, our analyses suggest its potential use. Force inference methods have the potential to advance cell evaluation techniques in biomedicine.

## Supporting information

S1 Data Simulation results of homogeneous cells. Simulation results of homogeneous cells, based on different parameters, are stored in approximately 400 folders, each individually named from “data_004” to “data_400”. Please refer to the Excel file “parameter_settings.xlsx” for detailed parameter settings. Each folder contains one.*dat* file representing the cell structure, two.*txt* files listing the correct stress values, and two.*vtk* files containing the estimated stress values. If a folder is empty, it means that the simulation was stopped for some reason under the parameters. Simulation results of heterogeneous cells. Simulation results of heterogeneous cells, based on different parameters, are stored in four folders, each individually named from “data_1001” to “data_1004”. The Excel file “parameter_settings.xlsx” also contains the detailed parameter settings of those four results. Each folder contains five files, arranged in the same configuration as above.
(ZIP)
